# Nox2-dependent Neuroinflammation in An EAE Model of Multiple Sclerosis

**DOI:** 10.1515/tnsci-2019-0001

**Published:** 2019-03-26

**Authors:** Katherine G. Ravelli, Graziella D. Santos, Nilton B. dos Santos, Carolina D. Munhoz, Deborah Azzi-Nogueira, Ana Carolina Campos, Rosana L. Pagano, Luiz R. Britto, Marina S. Hernandes

**Affiliations:** 1 Department of Physiology and Biophysics, University of São Paulo, São Paulo, Brazil; 2 Department of Pharmacology, University of São Paulo, São Paulo, Brazil; 3 Division of Cardiology, Department of Medicine Emory University, Atlanta, GA, United States; 4 Laboratory of Neuroscience, Hospital Sirio-Libanes, Sao Paulo, SP, Brazil

**Keywords:** Multiple sclerosist, NADPH oxidaset, astrocytest, cytokines

## Abstract

**Background:**

Multiple sclerosis (MS) is an inflammatory disease of the CNS, characterized by demyelination, focal inflammatory infiltrates and axonal damage. Oxidative stress has been linked to MS pathology. Previous studies have suggested the involvement of NADPH oxidase 2 (Nox2), an enzyme that catalyzes the reduction of oxygen to produce reactive oxygen species, in the MS pathogenesis. The mechanisms of Nox2 activation on MS are unknown. The purpose of this study was to investigate the effect of Nox2 deletion on experimental autoimmune encephalomyelitis (EAE) onset and severity, on astrocyte activation as well as on pro-inflammatory and anti-inflammatory cytokine induction in striatum and motor cortex.

**Methodology:**

Subcutaneous injection of MOG35-55 emulsified with complete Freund’s adjuvant was used to evaluate the effect of Nox2 depletion on EAE-induced encephalopathy. Striatum and motor cortices were isolated and evaluated by immunoblotting and RT-PCR.

**Results:**

Nox2 deletion resulted in clinical improvement of the disease and prevented astrocyte activation following EAE induction. Nox2 deletion prevented EAE-induced induction of pro-inflammatory cytokines and stimulated the expression of the anti-inflammatory cytokines IL-4 and IL-10.

**Conclusions:**

Our data suggest that Nox2 is involved on the EAE pathogenesis. IL-4 and IL-10 are likely to be involved on the protective mechanism observed following Nox2 deletion.

## Introduction

Multiple sclerosis (MS) is an autoimmune inflammatory disease of the central nervous system (CNS), characterized by demyelination, focal inflammatory infiltrates and axonal damage [1,2]. Several brain areas are affected, including but not limited to the cerebellum, brainstem, optic nerves and spinal cord [2]. Studies revealed atrophy of the temporal and frontal cortical areas, as well as demyelination in the thalamus, basal ganglia, hypothalamus, hippocampus, cerebellum and neocortex [3]. MS clinical symptoms include sensory or motor impairment, ataxia, spasticity, fatigue, and cognitive impairment [3].

Experimental autoimmune encephalomyelitis (EAE) is the most commonly used experimental model for MS induction [4]. EAE can be induced in rodents by immunization with myelin constituents, such as myelin basic protein, myelin oligodendrocyte glycoprotein (MOG) and proteolipid protein [5]. A subcutaneous injection of the antigen in complete Freund’s adjuvant (CFA) followed by two intraperitoneal injections of pertussis toxin (on the day of immunization and two days later) induces activation of myelin-specific T lymphocytes in the periphery and its migration into the CNS where T cells are reactivated by antigen-presenting cells leading to a subsequent inflammatory cascade and, eventually, demyelination and axonal degeneration [6].

Following EAE induction, activated macrophages and microglial cells produce multiple mediators of tissue damage, including proteases, nitric oxide (NO) and reactive oxygen species (ROS), resulting in neurodegeneration [7]. ROS generated by macrophages appear to be involved in demyelination and axonal damage induced by EAE [8]. ROS derived from mononuclear cells mediate oxidation of total DNA as well as mitochondrial DNA in patients with MS [9,10]. Moreover, evidence of lipid peroxidation has been described in exhaled breath samples of MS patients [11]. Additional studies have shown decreased levels of antioxidant enzymes in blood and cerebrospinal fluid of MS patients [12, 13].

NADPH oxidases (Noxes) are a family of enzymes able to catalyze the production of ROS. The prototypical Nox, the Nox2 isoform, is composed of three cytosolic components (p47^phox^, p67^phox^ e p40^phox^) and two membrane subunits (gp91^phox^ e p22^phox^). After stimulation, p47^phox^ is phosphorylated and the cytosolic components translocate to the membrane, where they associate with the membrane components, forming a functional Nox complex which utilizes the reduction equivalents of NADPH to reduce oxygen to superoxide [14].

Nox inhibition by apocynin, a nonspecific pharmacological Nox2 inhibitor, reduced profoundly the clinical features and neuropathological changes associated with EAE in MOG35-55-induced EAE model [15]. Moreover, an *in vitro* study demonstrated that the treatment with NADPH oxidase inhibitors (diphenyleneiodonium or apocynin) prevented myelin phagocytosis by peritoneal macrophages [8]. Furthermore, Nox2 mRNA expression was found to be upregulated in spinal cord in both relapsing-remitting and chronic models of EAE [16]. In MS patients, Nox2 was shown to be upregulated in activated microglia found in demyelinating and chronic MS lesions in brain tissue [17]. In a different study, it has been shown that microglial cells express Nox2 in preactive MS lesions, suggesting that microglial Nox2-derived ROS might be involved in the pathogenesis of MS [18]. A study using p47^phox^ knockout mice showed that Noxes inhibit the macrophage-derived NO, a strong inhibitor of T cell proliferation, which could be involved in the resistance of these animals to active EAE induction [19].

Although it has been previously demonstrated that EAE increases Nox activity in the brain and spinal cord [20], the mechanisms of Nox2 activation on the MOG_35-55-_induced EAE remain largely unknown. Here, we investigated the effects of EAE induction on the protein expression of a Nox2 subunit, the effect of Nox2 deletion on EAE onset and severity, astrocyte activation as well as on pro inflammatory and anti-inflammatory cytokines induction in brain structures commonly affected by EAE. Striatum and motor cortex, brain regions involved in motor control, were our choices of study.

## Material and Methods

### Animals

Female ten week-old gp91^phox-/-^ mice (obtained from Jackson Laboratories, Maine, USA) (*n* = 50) in the C57BL/6 background were used for this study. Controls for gp91^phox-/-^ mice were C57BL/6 mice (gp91^phox+/+^ mice - obtained from Jackson Laboratories, Maine, USA, *n* = 50). Mice had free access to food and water and were maintained on a 12:12 h light–dark cycle. All protocols were approved by the Ethics Committee for Animal Research of the University of Sao Paulo and experimental procedures were performed in accordance with the guidelines of the Brazilian College for Animal Experimentation (COBEA) and the animal care guidelines of the National Institutes of Health (NIH).

### EAE induction and behavioral score

Mice were given a flank subcutaneous injection of 150 μg of MOG_35-55_ emulsified with complete Freund’s adjuvant (MOG/CFA) which contained 0.3 mg of heat-inactivated Mycobacterium tuberculosis (H37RA; Difco Laboratories). Each animal also received pertussis toxin (200ng i.p; List Biological Laboratories) on day 0 and day 2 post-immunization. Mice were monitored daily up to 20 days for signs of disease. Control mice were treated with PBS emulsified with CFA without antigen. The EAE clinical score was determined based on the following scale: 0, no disease; 1, limp tail or isolated weakness of gait without limp tail; 2, partial hind limb paralysis; 3, total hind limb or partial hind and front limb paralysis; 4, total hind leg and partial front leg paralysis; 5, moribund or dead animal. The animals were euthanized for analysis 20 days following EAE induction. All behavior testing was performed in a blinded fashion. Different groups of mice were used for immunohistochemistry, immunoblotting and real-time PCR assays.

### Immunohistochemistry

Mice were deeply anesthetized (ketamine, 100 mg/kg of body weight and xylazine, 16 mg/kg of body weight, i.m.) and perfused transcardially with 0.1 M phosphate buffered saline (PBS) followed by 4% paraformaldehyde in 0.1 M sodium phosphate buffer (PB, pH 7.4). Brains were then removed and post-fixed for 4 h in the same fixative and cryoprotected in a 30% sucrose solution for at least 48 h at 4°C. Coronal brain sections (30 μm) were obtained on a sliding microtome and stored at 4°C. Free-floating sections were incubated for 12-16 h using an antibody against p47phox (Chemicon, USA). Samples were then incubated with a secondary antibody in phosphate buffered saline (PBS) with 0.3% TritonX-100 for 2 h at room temperature. After washing, samples were incubated with an avidin–biotin–peroxidase complex (ABC Elite kit, Vector Labs., Burlingame, CA, USA) for 2 h at room temperature. Labeling was developed with 0.05% diaminobenzidine tetrahydrochloride. The sections were mounted on glass slides, dehydrated and coverslipped using Permount (Fisher, Pittsburg, PA, USA). The material was analyzed on a light microscope and digital images were acquired. For the motor cortex, the analysis was focused on an intermediate area of the primary motor cortex, between 1 and 2 mm rostral to the bregma. Regarding the striatum, the analysis was performed in the rostral striatum (between 1 and 2mm rostral to the bregma). Brain areas of interest were identified using a stereotaxic atlas and semi-quantitative image analysis was performed using ImageJ software (National Institutes of Health/USA). Immunostaining optical density was evaluated within 0.4 mm^2^ areas for each brain structure analyzed as previously described [21]. The resulting indexes for the groups were then compared and subjected to statistical analysis using Graphpad Prism 3.02 (GraphPad Software Inc., San Diego, CA, USA).

### Western blotting

Mice were sacrificed and the regions of interest were quickly collected, frozen in liquid nitrogen, and stored at −70 °C until use. The tissue was homogenized in extraction buffer (Tris, pH 7.4, 100 mM; sodium pyrophosphate 100 mM; sodium fluoride 100 mM; EDTA 10 mM, sodium orthovanadate 10 mM; PMSF 2 mM; aprotinin 0.01 mg/ml) and the homogenates were centrifuged for 15 min at 15,300 x g at 4°C. The protein concentration of the supernatant was determined using the Bradford method (Bio- Rad, CA, USA). The samples were stored in sample buffer (Tris/HCl 500 mM, pH 6.8; 10% of SDS, 0.25% of bromophenol Blue; 10% of 2-mercaptoethanol and 50% glycerol) at −70 °C until starting the assay. Thirty μg of protein were boiled for 5 min, applied to acrylamide SDS gels (Bio-Rad, CA, USA) and electrophoretically transferred to nitrocellulose membranes (Millipore, Billerica, MA, USA) at 100V for 90 min. The membranes were then blocked for 1 hour at room temperature with PBS containing 0.05% Tween-20 (TTBS) and 5% nonfat milk and incubated overnight at 4^0^C with a monoclonal antibody anti-rabbit Gfap (1:1000; Santa Cruz Biotechnology, USA) diluted in TTBS with 1% non-fat milk. Following incubation with the appropriate HRP-conjugated secondary antibody, the probed proteins were developed by using a chemiluminescent kit (ECL, Amersham Biosciences, NJ, EUA). The target proteins were detected using a C-DiGit Western blot scanner (LI-COR, USA) and β-actin was used as an internal control. The quantification of band intensity was performed with ImageJ (National Institutes of Health, USA).

### Real- time PCR

Tissue from the brain regions of interest was collected, homogenized in 1 ml TRIzol (Invitrogen, Carlsbad, CA, USA) and total RNA was isolated following the manufacturer’s protocol. Briefly, following one chloroform extraction step, RNA was precipitated with isopropanol and the pellet washed once in 70% ethanol. After air-drying, the RNA was resuspended in DEPC-treated water and the concentration of each sample was obtained from A260/A280 nm measurements. One microgram total RNA was reverse transcribed by using the Promega Reverse Transcription System (Madison, WI, USA). Total RNA was incubated at 70°C for 10 minutes. The solution was mixed with 4μL of MgCl2 (25 mM), 2 μL of 5× first strand buffer, 2 μL of dNTP mixture (10 mM), 0.5 μl of RNAsin inhibitor (40U/μl), 0.5 μl of AMV reverse transcriptase and 1 μL of oligodT primer (0.5 μg). The reaction was incubated at 16°C for 30 min, 42°C for 30 min, 85°C for 5 min and then kept at 4°C. qPCR was carried out with SYBR Green Real-Time Selected Master Mix (Applied Biosystems, CA, USA) following the manufacturer’s protocol. The reaction mix (20 μL final volume) included the following: 2 μL diluted cDNA, 10 μL of SYBR Master Mix, and 500 nM of each primer. Amplification and PCR product detection were performed with the ABI prism 7500 real time-PCR System (Applied Biosystems, USA). PCR cycling conditions were set as follows: 50°C for 2 min, 95 °C for 2 min, then 30 cycles of 95°C for 15 s, 60°C for 1 min, and 72°C for 15 s. The specificity of the SYBR® green assay was confirmed by melting-point analysis. Expression data was calculated from the cycle threshold (Ct) value using the ΔCt method for quantification [22]. Primer sequences are indicated in Table 1.

**Table 1:** Description of primers used throughout the study to analyze the genes of interest

Gene	Primers sequence (5’ to 3’)	Amplicon length (bp)
IL-1β	FW: TGCCACCTTTTGACAGTGATG RV: ATGTGCTGCTGCGAGATTTG	136
MCP-1	FW: GGCTGGAGAGCTACAAGAGG	69
	RV: CTTGGTGACAAAAACTACAGC	
IL-4	FW: CAGCAACGAAGAACACCACAG RV: AAGCCCGAAAGAGTCTCTGC	144
IL-10	FW: GCGCTGTCATCGATTTCTCC	60
	RV: CTCTTCACCTGCTCCACTGC	
IL-6	FW: TCCTCTCTGCAAGAGACTTCC	80
	RV: TTGTGAAGTAGGGAAGGCCG	
GAPDH	FW: GTGCAGTGCCAGCCTCGTCC	107
	RV: CAGGCGCCCAATACGGCCAA	

IL-1β: interleukin 1 beta; MCP-1: monocyte chemoattractant protein -1; IL-4: interleukin 4; IL-10: interleukin 10; IL-6: interleukin 6; GAPDH: glyceraldehyde 3-phosphate dehydrogenase.

### Cytokine ELISAs

IL-4, IL-6 and IL-10 levels in brain tissue were measured using specific ELISA kits according to the manufacturer’s instructions (R&D Systems).

### Statistical analysis

Data are expressed as the mean ± SEM. For individual comparisons, statistical analysis was performed using unpaired Student’s *t*-test. Statistical analysis for EAE–induced changes in Nox2^+/+^ and Nox2^-/-^ mice were performed by one-way analysis of variance (ANOVA), followed by pairwise comparisons (Tukey’s HSD test). For the clinical score evaluation, a two-way ANOVA followed by a Bonferroni post hoc test was used to assess significance between Nox2^+/+^ and Nox2^-/-^ groups. In all cases, p ≤ 0.05 was considered to be statistically significant. Statistical analyses of data were generated using GraphPad Prism, version 3.02 (GraphPad Software Inc., San Diego, CA, USA).

## Results

### Effect of EAE on p47phox expression

**Figure 1:**
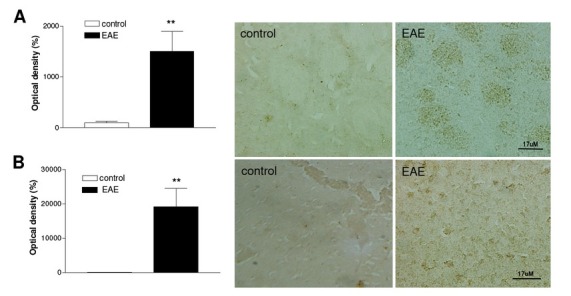
Effect of EAE induction on p47^phox^ immunoreactivity in striatum (A) and motor cortex (B) of Nox2^+/+^ mice. The graph depicts the mean optical density of p47^phox^ immunostaining. Representative digital images of p47^phox^-like immunoreactivity. **p<0.01 (Student’s *t*-test). N=4. EAE: Experimental autoimmune encephalomyelitis.

In both structures analyzed p47^phox^, a Nox2 cytosolic component, was found very diffuse and with a punctuate pattern of staining. There was a statistically significant increase in the expression of p47^phox^ in striatum (Figure 1A, p<0.01) and motor cortex (Figure 1B, p<0.01) of Nox2^+/+^ mice following EAE induction when compared to the control groups.

### Effect of Nox2 deletion on EAE onset and severity

**Figure 2:**
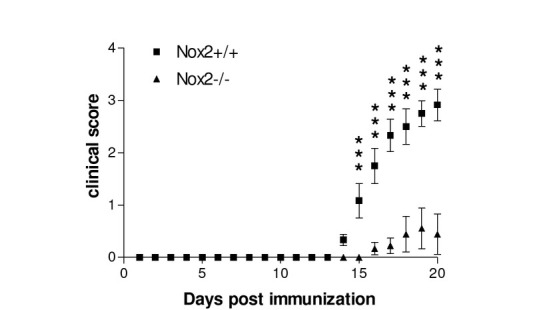
Effect of Nox2 deletion on EAE onset and severity. Nox2^+/+^ and Nox2^-/-^ mice were monitored daily up to 20 days for signs of disease following immunization. N=6-9. ***p<0.001 (Bonferroni’ s test).

As shown in Figure 2, in Nox2^+/+^ mice clinical signs of EAE were first noticed at day 14 post immunization and developed a rapid worsening that peaked around day 18 and were stable until day 20 post immunization. In Nox2^-/-^ mice, clinical symptoms were observed starting from day 16 and 17, and were overall less pronounced (p<0.001 versus daily clinical score for all days analyzed between days 15 and 20 post immunization) when compared to Nox2^+/+^ mice (Figure 2).

### Nox2 deletion abrogates GFAP protein expression induced by EAE

**Figure 3:**
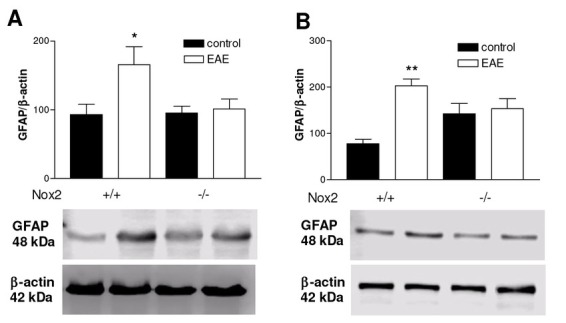
Effect of EAE on GFAP expression in striatum (A) and motor cortex (B) of Nox2^+/+^ and Nox2^-/-^ mice. Western blots analysis of the GFAP protein levels. The graphs represent mean ratio of GFAP densitometric data in relation to β-actin. *p<0.05 and **p<0.01 *vs* respective control (Tukey’s test). N=4-6. EAE: Experimental autoimmune encephalomyelitis.

Western blotting was used to quantitatively evaluate GFAP protein expression in the striatum and motor cortex following EAE induction. In both striatum and motor cortex, an increase in GFAP protein expression was observed in Nox2^+/+^ mice (p<0.05 and p<0.01, respectively), which was abrogated in Nox2^-/-^ mice (Figure 3).

### Nox2 mediates proinflammatory cytokine induction following EAE

**Figure 4:**
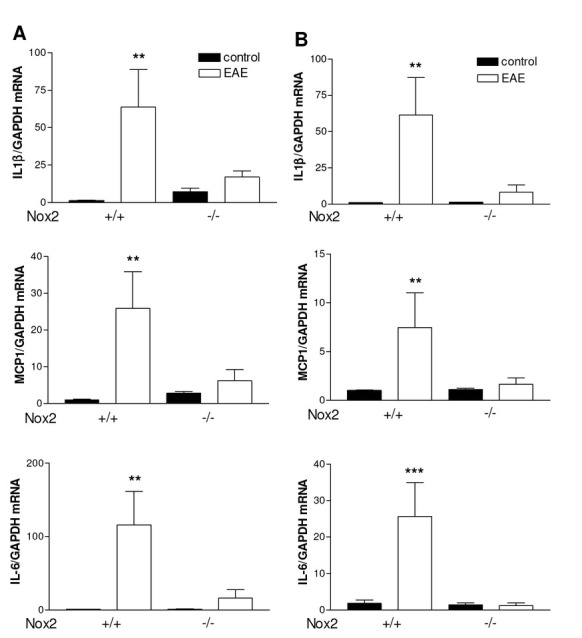
Effect of EAE induction on the mRNA levels of the pro-inflammatory cytokines IL-6, IL1β and MCP-1 in striatum (A) and motor cortex (B) of Nox2^+/+^ and Nox2^-/-^ mice. GAPDH was used as an internal control. **p<0.01 *vs* respective control, ***p < 0.001 *vs* respective control (Tukey’s test). N= 5-6. EAE: Experimental autoimmune encephalomyelitis.

To determine whether Nox2 regulates cytokine induction, we first examined their mRNA and expression levels after EAE induction. Twenty days following EAE induction, IL1β, MCP-1 and IL-6 mRNA expression were found significantly increased in both striatum (Figure 4A; p<0.01; p<0.01and p<0.01*vs* control, respectively) and motor cortex (Figure 4B; p<0.01; p<0.01 and p<0.001 *vs* control, respectively) of Nox2^+/+^ mice. Loss of Nox2 impaired EAE-induced IL1β, MCP-1 and IL-6 mRNA expression in both structures analyzed. Additionally IL-6 protein levels were evaluated in the brain tissue of Nox2^+/+^ and Nox2^-/-^ mice using ELISA. Although there was a trend toward significance between the Nox2^+/+^ EAE and the Nox2^+/+^ control groups, IL-6 protein levels were not significantly increased in the striatum of either Nox2^+/+^ and Nox2^-/-^ mice after EAE (Fig. 6). IL-6 protein expression was increased in the motor cortex of Nox2^+/+^ mice following EAE induction (Fig. 6; p<0.05 *vs* control); however, Nox2^-/-^ mice exhibited an attenuation of EAE-induced IL-6 expression compared to Nox2^+/+^ mice after EAE induction.

**Figure 5:**
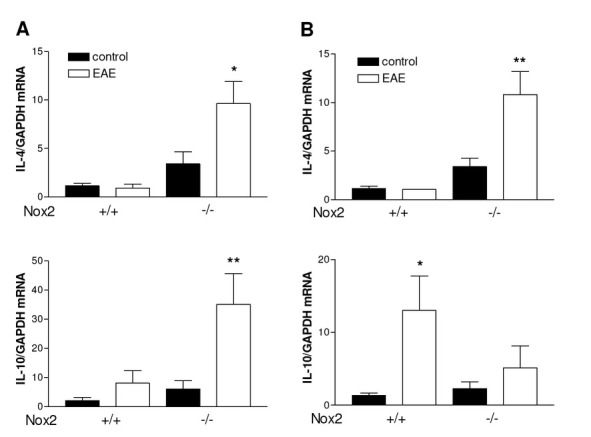
Effect of EAE induction on mRNA levels of the anti-inflammatory cytokines IL-4 and IL-10 in striatum (A) and motor cortex (B) of Nox2^+/+^ and Nox2^-/-^ mice. GAPDH was used as an internal control. *p<0.05 *vs* respective control, **p<0.01 *vs* respective control (Tukey’s test). N=5-6. EAE: Experimental autoimmune encephalomyelitis.

**Figure 6:**
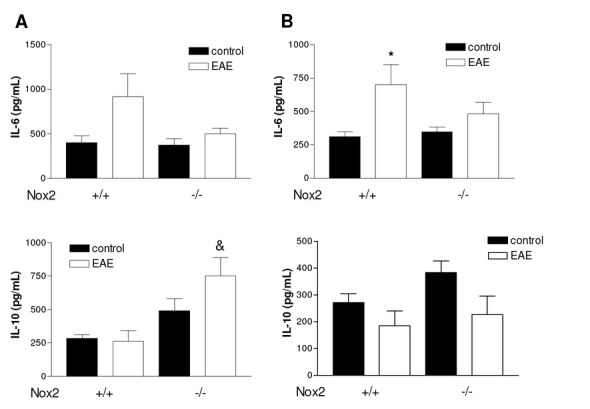
Effects of EAE induction on IL-6 and IL-10 protein levels in striatum (A) and motor cortex (B) of Nox2^+/+^ and Nox2^-/-^ mice. *p<0.05 and **p<0.01 *vs* respective control, &p<0.05 *vs* Nox2^+/+^ EAE (Tukey’s test). N=4-5. EAE: Experimental autoimmune encephalomyelitis.

### Effect of Nox2 deletion on the induction of anti inflammatory cytokines following EAE

In order to determine the effect of Nox2 deletion on the mRNA expression of the anti inflammatory cytokines IL-4 and IL-10 RT-PCR was performed. As shown in Figure 5, EAE induction did not increase IL-4 mRNA expression in either striatum or motor cortex of Nox2^+/+^ mice, however, loss of Nox2 significantly increased EAE-induced IL-4 in both structures analyzed (Figure 5A; p<0.05 *vs* Nox2^-/-^ control in striatum; Figure 5B; p<0.01 *vs* Nox2^-/-^ control in motor cortex). Similarly, IL-10 was found upregulated in the striatum of Nox2^-/-^ (Figure 5A; p<0.01 *vs* Nox2^-/-^ control) but not in Nox2^+/+^ mice following EAE induction. Conversely, in the motor cortex EAE induction was able to increase IL-10 mRNA expression only in Nox2^+/+^ (Figure 5B; p<0.05 *vs* Nox2^+/+^ control) but not in Nox2^-/-^ mice. Additionally, IL-4 and IL-10 protein levels were evaluated in in the striatum and motor cortex of Nox2^+/+^ and Nox2^-/-^ mice using ELISAs. IL-4 induction was found below detectable levels in both structures analyzed. Corroborating the mRNA expression data, IL-10 protein levels were found increased in the striatum of Nox2^-/-^ mice after EAE but not in the motor cortex (Figure 6, p<0.05 *vs* Nox2^+/+^ EAE).

## Discussion

In the present study, we present evidence to support our hypothesis that Nox2 plays an important role on the neuroinflammation induced by EAE. Our findings suggest that (1) p47^phox^ immunoreactivity is increased in striatum and motor cortex following EAE induction; (2) Nox2 deletion resulted in clinical improvement of the disease and prevented astrocyte activation as well as increased mRNA expression of proinflammatory cytokines following EAE induction (3) Nox2 deletion increased the induction of the antinflammatory cytokines IL-4 and IL-10 following EAE induction.

The human Nox2 gene is associated with MS and its expression is correlated with disease severity [23]. As noted earlier, p47^phox^ is among the most important subunits regulating Nox2 activity. We found a substantial increase in p47^phox^ immunoreactivity in both structures analyzed following EAE induction, suggesting the activation of Nox2 isoform. Of note, since Nox1 is also activated by forming a complex with p47^phox^ in a similar manner to Nox2 [24], we cannot rule out the possibility that p47^phox^ may also influence Nox1-specific signaling. Corroborating our results, Nox2 mRNA expression was found upregulated in spinal cord [25] and brain tissue [26] after EAE induction. Moreover, gene expression of the subunits p47^phox^, p67^phox^, and gp91^phox^, were all found increased after MOG-induced EAE. The Nuclear factor-erythroid 2–related factor 2 (Nrf2), a transcription factor that regulates genetic expression of many protective antioxidant enzymes, can be responsible for this neuroinflammatory response, since all these subunits were also significantly increased in Nrf2 knockout mice compared to the WT mice following EAE induction [27].

Nox2 has been implicated in several neuropathological conditions [26,28]. We have recently shown that Nox2 deletion protects mice against cognitive impairment induced by streptozotocin [28]. In the present study, Nox2 deletion significantly decreased clinical signs of EAE induced by the treatment with MOG35-55. In agreement with our data, Nox2 knockdown improved clinical scores, prevented body weight loss and oxidative stress-induced nitrotyrosine formation in a mouse model of EAE [29]. It has been demonstrated that Nox2 alters the pattern of proteolytic digestion. Nox2 depletion affected the ability of macrophages to process and present the I-Ab–immunodominant peptide of the MOG_35–55_. P47^phox^ and gp91^phox^ deficient mice were partially protected from MOG-induced EAE, partially due to an ineffective reactivation of MOG-specific CD4+ T cells in the CNS due to the inefficient processing of endogenous MOG to the MOG_35–55_ epitope by Nox2^-/-^ macrophages [30].

Astrocytes play a critical role in MS progression. They have been shown to inhibit remyelination and axonal regeneration by releasing several cytotoxic factors and also by forming a glial scar [31]. MS patients exhibited increased expression of GFAP in MS lesions [32] and in the cerebrospinal fluid [33]. Similarly, up-regulation of GFAP has been described in the spinal cord of mice in the chronic phase of EAE induced by MOG_35-55_ [33]. In our study we observed that EAE induction increased GFAP expression in striatum and motor cortex. Nox2 deletion attenuated GFAP immunoreactivity in both structures, suggesting an important role for Nox2 in mediating MOG_35-55_-induced astrocyte activation. Similar results have been described previously in other neurodegenerative conditions, such as Parkinson’s disease [21], sepsis induced encephalopathy [34] and Alzheimer’s disease [28].

The clear effect of Nox2 deletion on astrocyte activation suggested that the inflammatory response would be affected. The mRNA expression of IL-6, IL1β and MCP-1 was evaluated in striatum and motor cortex following EAE induction. The data indicate that the mRNA expression of all the cytokines analyzed was found significantly increased in both striatum and motor cortex of Nox2^+/+^ mice. Importantly, in our study, Nox2 deletion inhibited EAE-induced IL-6, IL1β and MCP-1 mRNA expression. It has been previously demonstrated that the expression of IL-6 is increased in macrophages and astrocytes in MS [35]. Moreover, its concentration was found elevated in serum and CSF [36]. IL-6 blockade by the treatment with an anti-IL-6 receptor monoclonal antibody inhibited the development of EAE in mice [37]. Corroborating our data, elevated levels of IL-6 have been described in CSF [38] and in brain structures in experimental models of MS [39]. IL-1β has been shown to be released by monocytes, microglia, astrocytes and brain endothelial cells and it is involved in inflammatory reactions within the CNS. Similarly to IL-6, enhanced levels of IL-1β have been described to be increased in MS lesions [40]. Of note, the correlation between IL-6, IL-1β and Nox2 was demonstrated in a study involving traumatic brain injury (TBI). TBI significantly increased mRNA levels of IL-6 and IL-1β in in the cortex, which was attenuated upon Nox2 knockdown [41]. Nox2 deletion has also been shown to inhibit streptozotocin-induced IL1β production in the hippocampus [28].

MCP-1, a monocyte chemoattractant, is produced by several immune and nonimmune cells [42]. In MS, this chemokine has been demonstrated to be released from astrocytes and macrophages and it seems to play a role in the demyelination of the CNS [43]. Increased expression of MCP-1 has been detected in astrocytes in the spinal cord following EAE induction [44]. Diphenyliodonium, a non-specific inhibitor of Nox2, inhibited MCP-1 release induced by LPS treatment in mice [45].

IL-10, a cytokine with potent anti-inflammatory and immunosuppressive activities, has been shown to be associated with both EAE [46] and MS remission [47]. In addition, several research groups have demonstrated that IFN-β treatment stimulates IL-10 production in MS, suggesting that IL-10 is involved in neuroprotection afforded by the treatment [48, 49, 50, 51]. On the basis of these reports, we hypothesized that the Nox2 knockdown would increase IL-10 induction, which is in fact what we observed. IL-10 was found upregulated in the striatum of Nox2^-/-^ mice at the protein and mRNA level, suggesting a role for IL-10 on the protective mechanism observed following Nox2 deletion. Similarly, Nox2 knockdown induced increased IL-10 mRNA expression in the cortex after TBI induction [52].

IL-4, another potent anti-inflammatory cytokine, is able to regulate the immune response, the production of proinflammatory cytokines and expression of major histocompatibility complex class II molecules [53]. The exogenous administration of IL-4 attenuates the disease severity after EAE induction [54]. Moreover, IL-4-deficient mice develop a more severe form of clinical disease, increased perivascular inflammation and demyelination, and increased mRNA expression of proinflammatory cytokines following EAE induction [55]. In the present study, EAE induction did not increase IL-4 expression in striatum or motor cortex of Nox2^+/+^ mice. However, Nox2 deletion significantly increased IL-4 expression in both brain structures after EAE induction. Our data suggests that IL-4 might also contribute to the improvement in clinical signs observed in MOG_35-55_-induced EAE in Nox2^-/-^ mice. Similar results were observed following LPS-induced neuroinflammation in p47^phox−/−^ mice. The brain levels of IL-4 as well as the mRNA expression of IL-4 receptor alpha were found increased after LPS injection in p47^phox−/−^ when compared to p47^phox+/+^ mice [56], suggesting a role for both Nox1 and Nox2 in LPS-induced neuroinflammation.

In summary, we have shown that Nox2 is involved in the MOG_35-55_-induced EAE model. Nox2 regulates cytokine mRNA levels and astrocyte activation *in vivo*. In addition, deletion of Nox2 leads to an improvement on EAE clinical signs and to a profound increase in the levels of the anti-inflammatory cytokines IL-4 and IL-10 in brain structures affected by the disease. Both IL-4 and IL-10 are likely to be involved on the protective mechanism observed following Nox2 deletion.

## Ethical approval

All procedures performed in studies involving animals were in accordance with the ethical standards of the University of Sao Paulo.

## Abbreviations

CFA: Complete Freund’s adjuvant
CNS: Central nervous system
EAE: Experimental autoimmune encephalomyelitis
MOG: Myelin oligodendrocyte glycoprotein
MS: Multiple sclerosis
NO: Nitric oxide
Nox2: NADPH oxidase 2
Noxes: NADPH oxidases
PBS: Phosphate buffered saline
ROS: Reactive oxygen species
